# Preoperative immune-inflammation index in predicting the diagnosis and adverse pathological features of prostate cancer

**DOI:** 10.3389/fonc.2025.1537542

**Published:** 2025-04-03

**Authors:** Chao-Long Liang, Wei-Da Li, Jian Wang

**Affiliations:** Department of Urology, Affiliated Hospital of Guangdong Medical University, First Clinical Medical College, Guangdong Medical University, Guangdong, China

**Keywords:** adverse pathological features, prostate cancer, retrospective study, inflammation, systemic immune-inflammation index (SII)

## Abstract

**Background:**

Studies have reported that the systemic immune-inflammation index (SII) is positively correlated with genitourinary cancers. This study aims to explore the predictive value of preoperative immune-inflammation index for the diagnosis of prostate cancer and its adverse clinical characteristics.

**Methods:**

This study analyzed patients who underwent their first prostate biopsy in the Urology Department of the Affiliated Hospital of Guangdong Medical University from January 2020 to January 2024. The predictive ability of SII for prostate cancer was evaluated, and the correlation between SII and localized prostate cancer and metastatic prostate cancer was explored.

**Results:**

The SII in the PCa group was significantly higher than in the BPH group (558.14 vs. 515.06, P = 0.022), and SII independently predicted PCa risk (OR = 1.001, P = 0.013). Metastatic PCa patients exhibited higher SII compared to localized cases (694.80 vs. 437.95, P < 0.001), with multivariate analysis confirming SII, neutrophils, lymphocytes, and PSA as independent predictors of metastasis (OR = 1.000, P = 0.044). ROC analysis revealed limited predictive power of SII alone (AUC = 0.559), but its combination with PSA significantly improved accuracy (AUC = 0.791). A comprehensive model integrating SII, age, uric acid, and PSA achieved an AUC of 0.823, outperforming PSA alone (AUC = 0.777).

**Conclusions:**

SII enhances the accuracy of PCa diagnosis and metastatic risk prediction when combined with PSA, demonstrating significant clinical utility. Although SII alone has limited predictive value, its cost-effectiveness and accessibility make it a valuable tool for stratified PCa management. Prospective studies are needed to validate its long-term prognostic significance.

## 1 Introduction

Prostate cancer (PCa) is a common malignancy of the urinary system (1). Over the past two decades, advancements in diagnostic and therapeutic strategies have significantly reduced the mortality risk associated with PCa. Prostate-specific antigen (PSA) has played a crucial role in early screening, enabling early detection and intervention to lower mortality rates (2, 3). However, it has become evident that PSA testing is not entirely reliable, as elevated PSA levels can also occur in benign conditions (2). Therefore, PSA alone lacks adequate sensitivity and specificity as an early diagnostic marker for PCa. Despite improvements with additional parameters such as PSA ratio, PSA density (PSAD), and the Prostate Imaging Reporting and Data System (PI-RADS) score, the diagnostic accuracy remains suboptimal. Furthermore, the prognosis for prostate cancer remains challenging due to local recurrence or distant metastasis. As a result, serum biomarkers are increasingly valued as non-invasive diagnostic tools due to their simplicity and predictive potential.

In 1863, the concept of “inflammation-cancer transformation” was first proposed. Virchow and colleagues discovered inflammatory infiltration within tumor tissues, suggesting the scientific hypothesis that cancer originates from chronic inflammation (4). In recent years, an increasing number of clinical studies have confirmed that the inflammatory process is involved in various stages of tumor development (5). Currently, a range of inflammatory markers, such as the neutrophil-to-lymphocyte ratio (NLR), platelet-to-lymphocyte ratio (PLR), and monocyte-to-lymphocyte ratio (MLR), have been validated in multiple studies for predicting treatment outcomes and prognosis in patients with various malignancies, including PCa (6–9). However, their diagnostic efficacy remains suboptimal. The Systemic Immune Inflammation Index (SII) is a newly discovered inflammation marker derived from the formula neutrophil count × platelet count/lymphocyte count. Compared to traditionally used hematological indicators such as NLR, PLR, and MLR, SII includes a broader range of immune cell parameters and has shown significant potential in predicting malignancies and their prognosis. Previous studies have primarily focused on traditional inflammatory markers such as NLR, with most research concentrating on end-stage metastatic castration-resistant prostate cancer (mCRPC). However, there has been a lack of studies on newly diagnosed metastatic hormone-sensitive prostate cancer (mHSPC). In 2024, Wang et al. (10) conducted a study that expanded upon previous research by incorporating additional immune-inflammatory markers, including SII, the lung immune prognostic index (LIPI), and the systemic inflammation response index (SIRI). Their findings demonstrated the prognostic value of SII, SIRI, and LIPI in both mHSPC and mCRPC, with LIPI exhibiting a more significant discriminative ability in the mHSPC stage. Building upon these insights, the present study, utilizing a larger sample size, explores the correlation between SII and PCa, with a particular focus on its predictive value for adverse pathological features. Furthermore, this study validates the potential of SII as a supplementary tool to PSA in clinical practice. In recent years, clinical studies on the relationship between SII and prostate cancer have yielded conflicting conclusions. Thus, there remains considerable controversy regarding the predictive value of SII for prostate cancer and adverse pathological features, necessitating further in-depth research.

In conclusion, inflammation is inextricably linked to tumorigenesis, invasion, and metastasis. Identifying specific anti-inflammatory targets is one of the key directions for the prevention and treatment of PCa. However, studies on the association between SII and PCa remain limited and controversial. Therefore, this study aims to analyze the clinical and pathological data of patients undergoing prostate biopsy at our center to investigate the clinical significance of SII in PCa diagnosis. Additionally, it explores the correlation between SII and pathological diagnosis as well as adverse clinical features of PCa, assessing its potential prognostic value. Ultimately, this study seeks to provide valuable insights for the early diagnosis and treatment of PCa in clinical practice.

## 2 Methods

### 2.1 Patients

This study retrospectively included 526 patients who underwent transrectal ultrasound-guided cognitive fusion prostate biopsy in the Department of Urology at the Affiliated Hospital of Guangdong Medical University from January 2020 to January 2024. All biopsies were performed by senior urologists in the department, and the pathological results were provided by the Pathology Department of the hospital.

Inclusion Criteria for Study Population: Age >18 years. Suspected prostate cancer patients who underwent initial prostate biopsy in the Department of Urology at our hospital between January 2020 and January 2024. Complete clinical and pathological data available. Exclusion Criteria: Patients with confirmed malignancies in other locations. History of prostate biopsy or surgery, or histopathological diagnosis of acute prostatitis, prostatic intraepithelial neoplasia, or atypical small acinar proliferation. Recent active infections or inflammatory diseases. Recent use of hormonal or anti-inflammatory medications. Recent history of blood transfusion or administration of blood products. Previous treatments with radiotherapy, chemotherapy, or immunotherapy. Presence of hematological, rheumatic, or immune system diseases, or other clinical conditions that could affect inflammation parameters (e.g., chronic hepatitis, liver failure).

Grouping Criteria: (1) All enrolled patients were divided into two groups based on the histopathological results of the prostate biopsy: Benign Prostatic Hyperplasia (BPH) Group: Patients diagnosed with BPH via biopsy. PCa Group: Patients diagnosed with PCa via biopsy. (2) All prostate cancer cases were further divided into two groups: Localized Prostate Cancer Group: Tumor confined within the prostate capsule or extending beyond the capsule to involve the seminal vesicles, sphincter, rectum, levator ani muscle, pelvic wall, or other adjacent structures (11). Metastatic Prostate Cancer Group: Tumor no longer confined to the prostate, with cancer cells metastasizing to other locations. Prostate cancer primarily spreads to regional lymph nodes and bones but may also metastasize to the liver, lungs, peritoneum, adrenal glands, and brain (12).

Based on Article 39(1)(12) of the 2016 “Ethical Review Methods for Biomedical Research Involving Humans” in China, this study does not require informed consent. The study has been approved by the Ethics Committee of the Institute and conducted in accordance with the Declaration of Helsinki (Ethics Approval No.:LY2024-07-012).

### 2.2 Data collection

All patient data were obtained from our hospital’s electronic medical record system. The collected study data included: demographic information (age, height, weight); preoperative peripheral blood test results (uric acid, white blood cells, neutrophils, lymphocytes, monocytes, hemoglobin, red blood cell distribution width, platelets, platelet distribution width, serum prostate-specific antigen); imaging examination results (color Doppler ultrasound, computed tomography, magnetic resonance imaging); and biopsy pathology results.

### 2.3 Statistical analysis

The experimental data were analyzed using SPSS 26.0 and GraphPad Prism 5 software for data analysis and graph creation. Normally distributed measurement data were expressed as mean ± standard deviation, and independent sample t-tests were used to compare the indicators between the two groups. Non-normally distributed measurement data were expressed as median (interquartile range) and analyzed using the Wilcoxon rank-sum test to examine differences between groups. Categorical data were presented as frequency and percentage (%), and comparisons were made using the chi-square test. Univariate analysis was performed to evaluate each variable individually, and variables with statistical significance were included in a multivariate logistic regression analysis to identify independent factors associated with the outcomes. The area under the curve (AUC) of each indicator was calculated using receiver operating characteristic (ROC) curves to evaluate the predictive performance of each indicator. A two-sided test was used for significance testing, with P<0.05 considered statistically significant.

## 3 Results

### 3.1 Analysis of patients with benign prostatic hyperplasia and patients with prostate cancer

A total of 526 patients were included in the study, with a median age of 71.00 years (65.00, 77.25 years). The mean BMI was 22.24 ± 3.32 kg/m². The median systemic immune-inflammation index (SII) was 532.05 (351.85, 831.77). The median uric acid level was 370.60 (311.60, 426.85) μmol/L. The median lymphocyte count was 1.59 (1.25, 2.02). The median hemoglobin (Hb) level was 131.00 (118.00, 142.00) g/L. The median platelet distribution width (PDW) was 10.60 (9.70, 11.80)%. The median PSA level was 22.89 (8.48, 75.44) ng/mL. Baseline characteristics of all patients are shown in **Table 1**.

**Table 1:** Clinical characteristics of patients with benign prostatic hyperplasia and patients with prostate cancer.

Variable	All	Prostatic hyperplasia patient Group	Prostate cancer patient Group	P-value
Cases	526	204	322	–
Age (years)	71.00 (65.00,77.25)	68.00 (62.00, 74.00)	74.00 (68.00, 84.00)	<0.001
BMI(kg/m²)	22.24 ± 3.32	22.69 ± 3.01	21.99 ± 3.46	0.024
SII	532.05 (351.85, 831.77)	515.06 (337.83, 710.35)	558.14 (361.29, 882.71)	0.022
Uric acid (μmol/L)	370.60 (311.60, 426.85)	356.40 (311.25, 408.48)	382.70 (316.20, 440.20)	0.025
White blood cell	6.67 (5.66, 8.09)	6.50 (5.61, 7.89)	6.80 (5.68, 8.47)	0.104
Neutrophils	4.05 (3.18, 5.26)	3.89 (3.20, 4.88)	4.18 (3.17, 5.45)	0.234
Lymphocyte	1.59 (1.25, 2.02)	1.72 (1.40, 2.10)	1.53 (1.18, 1.98)	<0.001
Monocyte	0.60 (0.48, 0.73)	0.59 (0.48, 0.70)	0.62 (0.48, 0.75)	0.089
Hb(g/L)	131.00 (118.00, 142.00)	133.00 (121.00, 145.00)	129.00 (115.00, 140.00)	0.003
RDW(%)	12.90 (12.40, 13.70)	12.80 (12.40, 13.78)	13.00 (12.40, 13.70)	0.186
PLT	217.50 (183.25, 255.00)	218.00 (187.25, 255.75)	216.00 (181.00, 254.00)	0.471
PDW(%)	10.60 (9.70, 11.80)	10.85 (9.80, 11.90)	10.40 (9.60, 11.70)	0.041
PSA(ng/mL)	22.89 (8.48, 75.44)	11.35 (6.37, 21.76)	48.76 (15.14, 162.40)	<0.001

Based on the biopsy results, the 526 patients were divided into two groups: the benign BPH group, with 204 patients (38.8%), and the prostate cancer group, with 322 patients (61.2%), as shown in **Table 1**. Comparative analysis between the two groups indicated that patients in the prostate cancer group had higher age, SII, uric acid, and PSA levels compared to the BPH group, whereas BMI, lymphocyte count, hemoglobin, and PDW were lower in the prostate cancer group. These differences were statistically significant (P<0.05). Specifically, the median SII was 515.06 (337.83, 710.35) in the BPH group and 558.14 (361.29, 882.71) in the prostate cancer group.

The results of the univariate and multivariate logistic regression analyses are shown in **Table 2**. Variables that showed statistically significant differences between the two groups were individually included in the univariate logistic regression analysis. The results indicated that prostate cancer was associated with age (OR: 1.074, 95% CI: 1.050–1.097, p < 0.001), BMI (OR: 0.938, 95% CI: 0.886–0.994, p = 0.030), SII (OR: 1.001, 95% CI: 1.000–1.001, p < 0.001), uric acid (OR: 1.002, 95% CI: 1.000–1.004, p = 0.044), lymphocyte count (OR: 0.638, 95% CI: 0.481–0.848, p = 0.002), hemoglobin (OR: 0.982, 95% CI: 0.973–0.992, p < 0.001), and PSA (OR: 1.034, 95% CI: 1.024–1.044, p < 0.001), but not with PDW.

**Table 2:** Univariate and multivariate logistic regression analysis of patients with benign prostatic hyperplasia and patients with prostate cancer.

Variable	Univariate analysis		Multivariate analysis	
	OR (95%CI)	P-value	OR (95%CI)	P-value
Age	1.074 (1.050,1.097)	<0.001	1.052 (1.022,1.084)	0.001
BMI	0.938 (0.886,0.994)	0.030	1.002 (0.927,1.082)	0.963
SII	1.001 (1.000,1.001)	<0.001	1.001 (1.000,1.001)	0.013
Uric acid	1.002 (1.000,1.004)	0.044	1.003 (1.000,1.006)	0.020
Lymphocyte	0.638 (0.481,0.848)	0.002	1.240 (0.790,1.946)	0.349
Hb	0.982 (0.973,0.992)	<0.001	0.999 (0.986,1.012)	0.869
PDW	0.955 (0.875,1.043)	0.310		
PSA	1.034 (1.024,1.044)	<0.001	1.034 (1.022,1.046)	<0.001

After including the significantly associated variables from the univariate regression analysis into the multivariate regression analysis, the results showed that age (OR: 1.052, 95% CI: 1.022–1.084, p = 0.001), SII (OR: 1.001, 95% CI: 1.000–1.001, p = 0.013), uric acid (OR: 1.003, 95% CI: 1.000–1.006, p = 0.020), and PSA (OR: 1.034, 95% CI: 1.022–1.004, p < 0.001) are independent risk factors for prostate cancer.

**Figure 1:**
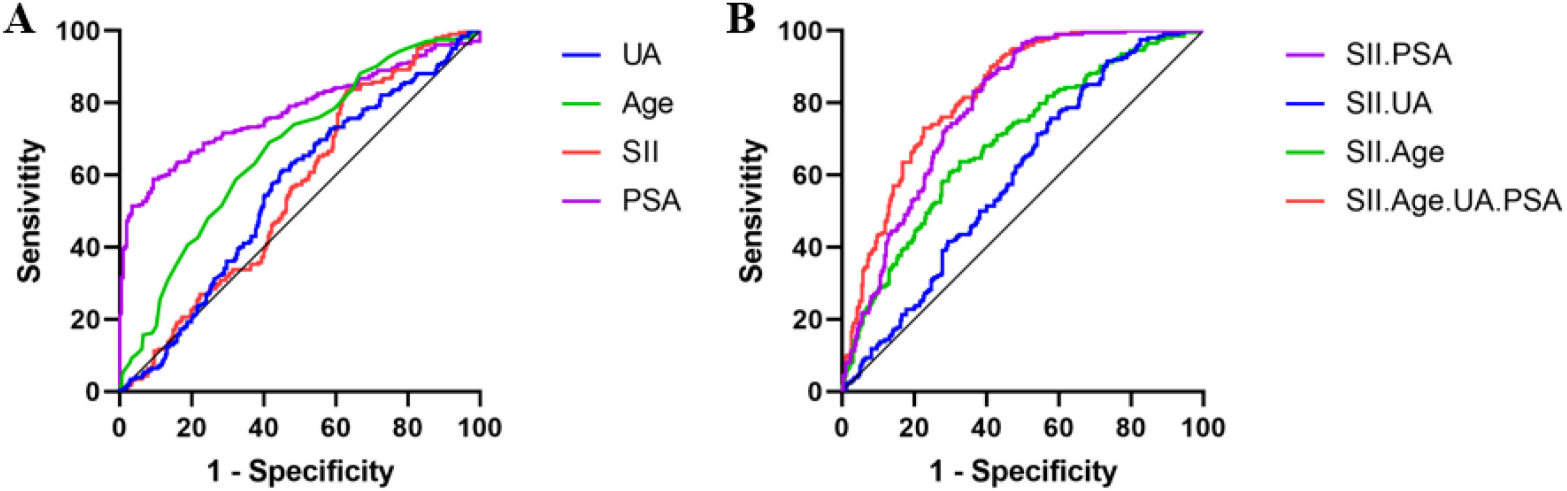
ROC Curves for Individual and Combined Predictions of Prostate Cancer by Each Independent Risk Factor. **(A)**. ROC Curves for Individual Predictions by Each Independent Risk Factor. **(B)**. ROC Curves for Predictions Combined with SII and Each Independent Risk Factor.

**Figure 1A** shows the individual predictions of prostate cancer by each independent risk factor. ROC analysis results indicated that the area under the curve (AUC) for PSA was 0.777 (95% CI: 0.737-0.816, P < 0.001), for age was 0.672 (95% CI: 0.626-0.719, P < 0.001), for SII was 0.559 (95% CI: 0.510-0.608, P = 0.022), and for uric acid was 0.558 (95% CI: 0.508-0.608, P = 0.025).

**Figure 1B** illustrates the prediction of prostate cancer by combining SII with each independent risk factor. ROC analysis results indicated that the combined AUC for SII and PSA was 0.791 (95% CI: 0.752-0.828, P < 0.001), for SII and uric acid was 0.596 (95% CI: 0.547-0.644, P < 0.001), for SII and age was 0.690 (95% CI: 0.643-0.736, P < 0.001), and for SII combined with age, uric acid, and PSA was 0.823 (95% CI: 0.788-0.858, P < 0.001).

### 3.2 Analysis of localized prostate cancer patient group and metastatic prostate cancer patient group

According to the 2022 AUA/ASTRO clinical guidelines for localized prostate cancer, patients diagnosed with prostate cancer via biopsy were categorized into two groups: localized prostate cancer patient group and metastatic prostate cancer patient group. The localized prostate cancer group comprised 158 cases (49.07%), while the metastatic prostate cancer group comprised 164 cases (50.93%), as shown in **Table 3**. Comparative analysis between the two groups revealed that patients in the metastatic prostate cancer group had higher SII, neutrophil count, monocyte count, and PSA levels compared to the localized prostate cancer group. Conversely, BMI, lymphocyte count, hemoglobin, and PDW were lower in the metastatic prostate cancer group compared to the localized prostate cancer group. These differences were statistically significant (P < 0.05). Specifically, the median SII was 437.95 (315.59, 777.20) in the localized prostate cancer group and 694.80 (409.44, 988.81) in the metastatic prostate cancer group.

**Table 3:** Clinical characteristics of localized prostate cancer patient group and metastatic prostate cancer patient group.

Variable	Prostate cancer patient Group	Localized prostate cancer Group	Metastatic prostate cancer Group	P-value
Cases	322	158	164	–
Age	74.00 (68.00, 84.00)	73.00 (65.75, 80.00)	74.00 (69.00, 80.00)	0.127
BMI (kg/m²)	21.99 ± 3.46	22.44 ± 3.57	21.55 ± 3.31	0.027
SII	558.14 (361.29, 882.71)	437.95 (315.59, 777.20)	694.80 (409.44, 988.81)	<0.001
Uric acid(μmol/L)	382.70 (316.20, 440.20)	377.30 (312.40, 441.15)	385.70 (319.50, 437.60)	0.881
White blood cell	6.80 (5.68, 8.47)	6.59 (5.69, 8.47)	7.13 (5.59, 8.58)	0.304
Neutrophils	4.18 (3.17, 5.45)	3.84 (3.05, 4.99)	4.45 (3.38, 5.55)	0.002
Lymphocyte	1.53 (1.18, 1.98)	1.66 (1.31, 2.19)	1.42 (1.10, 1.73)	<0.001
Monocyte	0.62 (0.48, 0.75)	0.58(0.46, 0.72)	0.65 (0.53, 0.78)	0.003
Hb(g/L)	129.00 (115.00, 140.00)	134.00 (119.75, 145.00)	123.00 (113.25, 134.00)	<0.001
RDW(%)	13.00 (12.40, 13.70)	13.00 (12.40, 13.43)	13.10 (12.43, 14.10)	0.108
PLT	216.00 (181.00, 254.00)	210.00 (179.50, 252.50)	221.00 (183.00, 256.50)	0.453
PDW(%)	10.40 (9.60, 11.70)	10.85 (9.80, 12.20)	10.00 (9.50, 11.10)	0.001
PSA(ng/mL)	48.76 (15.14, 162.40)	22.84 (9.45, 48.64)	112.10 (46.79, 447.08)	<0.001

As shown in **Table 4**, variables that showed a statistically significant difference between the localized prostate cancer group and the metastatic prostate cancer group, along with age, were individually included in univariate logistic regression analysis. The results indicated that prostate cancer was associated with BMI (OR: 0.927, 95% CI: 0.867–0.992, p=0.029), SII (OR: 1.001, 95% CI: 1.000–1.001, p=0.025), neutrophils (OR: 1.154, 95% CI: 1.034–1.287, p=0.011), lymphocytes (OR: 0.475, 95% CI: 0.325–0.696, p<0.001), monocytes (OR: 6.488, 95% CI: 2.272–18.530, p<0.001), Hb (OR: 0.970, 95% CI: 0.958–0.983, p<0.001), PDW (OR: 0.874, 95% CI: 0.781–0.978, p=0.019), and PSA (OR: 1.006, 95% CI: 1.004–1.009, p<0.001).

**Table 4:** Univariate and multivariate logistic regression analysis of localized and metastatic prostate cancer patient groups.

Variable	Univariate analysis		Multivariate analysis	
	OR (95%CI)	P-value	OR (95%CI)	P-value
Age	1.021 (0.995,1.047)	0.113	0.993 (0.956,1.030)	0.702
BMI	0.927 (0.867,0.992)	0.029	1.008 (0.921,1.104)	0.856
SII	1.001 (1.000,1.001)	0.025	1.000 (1.000,1.001)	0.044
Neutrophils	1.154 (1.034,1.287)	0.011	1.410 (1.065,1.867)	0.016
Lymphocyte	0.475 (0.325,0.696)	<0.001	0.331 (0.162,0.675)	0.002
Monocyte	6.488 (2.272,18.530)	<0.001	3.389 (0.633,18.142)	0.154
Hb	0.970 (0.958,0.983)	<0.001	0.978 (0.961,0.995)	0.011
PDW	0.874 (0.781,0.978)	0.019	0.764 (0.642,0.910)	0.002
PSA	1.006 (1.004,1.009)	<0.001	1.008 (1.005,1.012)	<0.001

The variables that were significantly associated in the univariate regression analysis, along with age, were included in the multivariate regression analysis. The results showed that SII (OR: 1.000, 95% CI: 1.000–1.001, p=0.044), neutrophils (OR: 1.410, 95% CI: 1.065–1.867, p=0.016), lymphocytes (OR: 0.331, 95% CI: 0.162–0.675, p=0.002), hemoglobin (OR: 0.978, 95% CI: 0.961–0.995, p=0.011), PDW (OR: 0.764, 95% CI: 0.642–0.910, p=0.002), and PSA (OR: 1.008, 95% CI: 1.005–1.012, p<0.001) were independent risk factors for metastatic prostate cancer.

**Figure 2:**
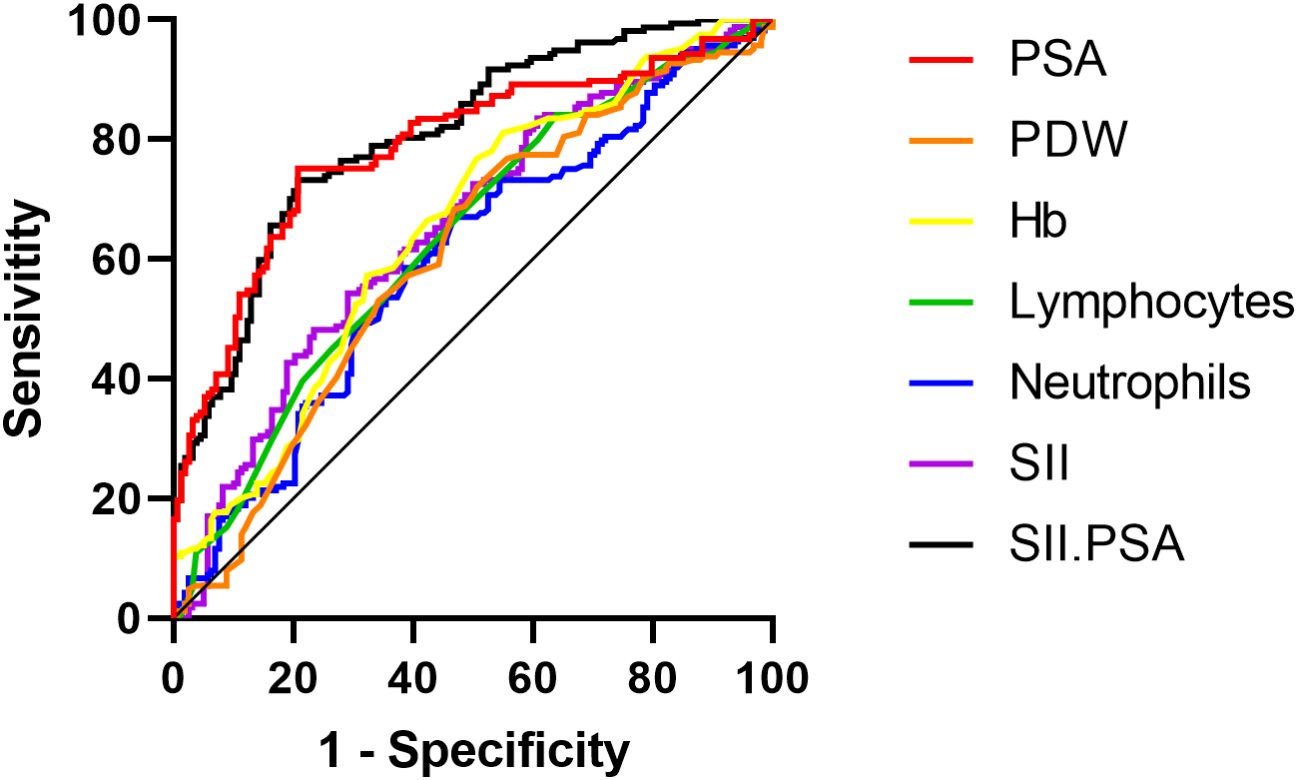
ROC curves for individual and combined independent risk factors predicting metastatic prostate cancer.

As shown in **Figure 2**, the ROC analysis results for predicting metastatic prostate cancer using individual independent risk factors are as follows: the area under the curve (AUC) for PSA is 0.788 (95% CI: 0.737-0.839, P<0.001), for PDW is 0.608 (95% CI: 0.546-0.670, P<0.001), for Hb is 0.652 (95% CI: 0.592-0.712, P<0.001), for lymphocytes is 0.633 (95% CI: 0.573-0.694, P<0.001), for neutrophils is 0.598 (95% CI: 0.536-0.660, P=0.023), for SII is 0.650 (95% CI: 0.590-0.710, P<0.001), and for the combination of SII and PSA is 0.806 (95% CI: 0.759-0.854, P<0.001).

## 4 Discussion

Currently, screening and auxiliary diagnosis of PCa primarily rely on PSA (2, 3). However, it has been gradually recognized that PSA can also be elevated to abnormal levels in many benign conditions (2). Numerous studies have reported on whether patients in the PSA “gray zone” require further biopsy, but there is still no definitive conclusion (13). Therefore, researchers are continuously exploring simpler, more effective, and less invasive indicators to evaluate and predict prostate cancer, thereby reducing unnecessary biopsies and the waste of medical resources.

In recent years, numerous studies have reported that immune-inflammatory cells in peripheral blood can drive tumorigenesis, growth, progression, and transformation (6, 7). Neutrophils promote tumorigenesis and progression through immunosuppression (8), angiogenesis (9), and metastasis (14). A low lymphocyte count is associated with poor clinical outcomes (15). Platelets aid cancer cells in evading immune surveillance and are involved in tumor proliferation and invasion (16). Based on the tumor microenvironment established by these peripheral blood cells, the value of SII in the diagnosis and prognosis of solid tumors such as breast cancer, pancreatic cancer, lung cancer, and cervical cancer has been reported (17–20). Additionally, meta-analyses have shown that SII also demonstrates certain diagnostic and prognostic value in various urological tumors such as prostate cancer, renal cell carcinoma, bladder cancer, and upper urinary tract urothelial carcinoma (21). Qi et al. (22) conducted a meta-analysis that included 10 studies related to PCa, primarily investigating the association between SII and metastatic prostate cancer (mPCa) as well as mCRPC. Their results indicated that elevated SII was associated with poor overall survival (OS) in mCRPC patients, as well as unfavorable biochemical recurrence-free survival and adverse pathological features in non-metastatic PCa (nmPCa) patients. The authors concluded that SII could serve as a prognostic predictor for PCa patients, with its application potentially enhancing the diagnosis and treatment of prostate cancer. However, in recent years, clinical studies on the relationship between SII and prostate cancer have yielded different conclusions. Another retrospective analysis conducted in 2020 indicated that SII did not have statistical significance for the survival rate of prostate cancer and suggested that further research is needed to effectively integrate inflammatory markers into prognostic models (23). Similarly, a prospective study by Murray et al. (24) included a total of 1,223 men, among whom 467 (38%) were diagnosed with PCa based on biopsy results. The study found no significant difference in SII between PCa patients and those with negative biopsy results. However, the PLR was significantly elevated in PCa patients. This discrepancy may be attributed to differences in sample population selection, inadequate control of inflammatory factors, and variations in SII cutoff values. In contrast, the present study, focusing on newly diagnosed PCa patients, constructed a predictive model and found that the median SII in the PCa group was 558.14 (361.29, 882.71)/L, which was higher than that in BPH group at 515.06 (351.85, 831.77)/L (P = 0.022). Moreover, multivariable logistic regression analysis identified SII as an independent risk factor for PCa (OR = 1.001, P < 0.001). Therefore, we believe that although there may not be a clear causal relationship between SII and PCa, there is a strong association, and SII can be considered to have certain reference value in predicting PCa. In the localized prostate cancer group, the median SII was 437.95 (315.59, 777.20)/L, whereas in the metastatic prostate cancer group, the median SII was 694.80 (409.44, 988.81)/L (P<0.001), indicating a significant difference between the two groups. High SII appears to have clinical significance in predicting adverse pathological features of prostate cancer or distinguishing metastatic prostate cancer, which is consistent with findings from studies on SII and other solid tumors. This further validates the potential of SII as a diagnostic and prognostic marker for PCa. Compared to traditional PSA testing, SII not only reflects the presence of the tumor but also reveals changes in the host immune status, providing a basis for a more comprehensive disease assessment. Therefore, our study results indicate that SII plays a crucial role in predicting prostate cancer and its adverse pathological features, and also demonstrate that the development and progression of prostate cancer are closely related to immune-inflammatory status. Currently, novel anti-tumor drugs targeting the immune system are under investigation and have reached the clinical trial stage, which further supports our findings. In summary, SII can aid clinicians in more accurately predicting the prognosis of prostate cancer patients, and these peripheral blood components may have increasing prognostic and potential value as therapeutic targets for prostate cancer in the future.

Serum PSA currently plays a crucial role in the early screening of prostate cancer and is a highly specific tumor marker. The results of this study demonstrate that serum PSA is an independent risk factor for prostate cancer, which is consistent with previous research [30, 31]. Compared to SII or other indicators, the differences in PSA levels between the prostate cancer group and the benign prostatic hyperplasia group are more pronounced, and PSA shows higher predictive efficacy for metastatic prostate cancer. In this study, the ROC curve for combined detection of SII, age, PSA, and uric acid in predicting prostate cancer had an AUC of 0.823, which is higher than that for PSA or SII alone. In differentiating between localized prostate cancer and metastatic prostate cancer, the ROC curve for the combined detection of SII and PSA had an AUC of 0.806, also higher than for PSA or SII alone. This indicates that while SII alone has a lower predictive efficacy for prostate cancer or metastatic prostate cancer compared to PSA, its predictive performance improves significantly when included in a model with PSA. Therefore, according to the results of this study, SII holds substantial potential as an adjunct to PSA in the diagnosis of prostate cancer. However, it is worth noting that the AUC for SII alone in detecting PCa and metastatic prostate cancer was 0.559 and 0.650, respectively. This indicates that SII has certain limitations when used independently, particularly in the early diagnosis of PCa, where its discriminatory ability is relatively low. Nevertheless, SII remains a potential biomarker for assisting in prostate cancer screening, especially in cases where other effective diagnostic methods are unavailable, providing additional assessment information for patients. As a comprehensive inflammatory marker, SII plays a role in the early diagnosis of prostate cancer, the evaluation of immune-inflammatory status, and prognosis prediction. Although its diagnostic performance is relatively low when used alone, combining SII with other biomarkers, such as PSA, may enhance the accuracy of prostate cancer diagnosis and improve the reliability of prognosis assessment. PSA, as the primary biomarker for PCa screening, showed an AUC of 0.777 in this study, which was higher than that of SII. However, SII still demonstrated some value in predicting metastatic PCa. When SII was combined with PSA, the AUC for predicting PCa increased to 0.791, suggesting that SII provides additional information on top of PSA testing. A prospective study included a total of 118 patients, among whom 73 received docetaxel as first-line treatment, 31 as second-line treatment, and 14 as third-line treatment. The results indicated that the modified Glasgow Prognostic Score (mGPS) before treatment may serve as a promising prognostic biomarker. The combination of mGPS with other inflammatory markers, such as SII, could provide optimized stratification for treatment selection (25). This further validates the significant potential of SII as an adjunct tool in guiding clinical decision-making for PCa. In the future, with further large-scale, multicenter clinical studies, SII may become an important tool in the screening and management of prostate cancer.

The immune-inflammatory status of the body influences PCa. However, SII does not encompass the intrinsic genetic characteristics of the tumor, which play a critical role in the onset, progression, and treatment response of PCa. Studies have revealed that Y chromosome loss may be associated with the development of PCa, and that genetic variations or deletions on the Y chromosome can induce T lymphocyte exhaustion, thereby impairing the normal immune function of leukocytes (26). Furthermore, research has found that the deletion of certain Y chromosome genes activates inflammation-related pathways (e.g., IL-6 and CCL2), promoting tumor development (27). Additionally, an animal study demonstrated that reintroducing the Y chromosome into PCa-bearing mice resulted in limited tumor suppression (28). The mechanisms by which Y chromosome loss and other genetic factors promote tumor growth are more complex than these findings suggest, but current research indicates that certain intrinsic genetic factors may be closely linked to systemic immune-inflammatory status and may even directly affect SII. Therefore, genomic testing after PCa diagnosis or surgery could provide valuable information for personalized treatment. In recent years, the use of genetic classifiers has become increasingly widespread. Some studies suggest that the trend of tissue-based genomic testing for PCa is on the rise, although the precise role of these tests in clinical practice remains a subject of ongoing debate (29). Combining SII with genetic classifiers could provide more comprehensive information for precision diagnosis and treatment of PCa. While SII can assess the systemic immune-inflammatory status of patients, genetic classifiers can provide information on the genetic risk of the tumor, thereby optimizing risk prediction models and enhancing the assessment of disease progression and treatment response. In the future, large-scale prospective studies should further validate the combined value of SII and genetic classifiers in PCa diagnosis and treatment, with the goal of offering clinicians more precise personalized treatment strategies.

In summary, the hematological indicators included in the SII are part of routine admission tests, making them relatively convenient and cost-effective. The results of this study show that SII is an independent factor for prostate cancer and also for metastatic prostate cancer. Combining SII with serum PSA can address the limitations of using SII or PSA alone, enhancing the diagnostic efficacy for both prostate cancer and metastatic prostate cancer, and providing valuable information for the early diagnosis and treatment of prostate cancer. Additionally, it offers more options for subsequent treatment strategies for prostate cancer. This study builds upon the research conducted by numerous scholars, which includes various types of solid tumors such as breast cancer and lung cancer. However, research specifically focusing on prostate cancer is relatively limited. Additionally, the data from this study center can contribute to expanding the prostate cancer database. Furthermore, the findings of this study may help optimize patient stratification and enable more personalized treatment approaches. Moreover, SII can be used in conjunction with existing biomarkers to provide a more comprehensive assessment of disease status or to enhance the diagnostic efficacy of current indicators.

Our study has several limitations. First, any retrospective data collection inherently carries limitations, particularly regarding potential selection bias. Second, some patients might have chronic inflammation, which could affect biomarker levels; however, patients with acute inflammation were excluded. Third, this study is a small-sample, single-center retrospective study. While it somewhat avoids potential differences in clinical practice and diagnosis seen in multi-center studies, selection bias inherent in such research cannot be entirely eliminated. Fourth, the limited follow-up time and lack of detailed data on recurrence, metastasis, and survival impede accurate prognostic survival assessment and affect the depth of the research results. Nevertheless, this study lays the groundwork for further exploration of the mechanisms underlying SII in prostate cancer and for designing more comprehensive prospective studies. Future research with larger sample sizes and clinical validation could position SII as a crucial auxiliary tool in prostate cancer diagnosis and treatment, enhancing diagnostic accuracy and prognostic evaluation.

## Data Availability

The original contributions presented in the study are included in the article/supplementary material. Further inquiries can be directed to the corresponding author.
